# Reliability of fetal nasal bone length measurement at 11–14 weeks of gestation

**DOI:** 10.1186/1471-2393-13-7

**Published:** 2013-01-16

**Authors:** Chitkasaem Suwanrath, Ninlapa Pruksanusak, Ounjai Kor-anantakul, Thitima Suntharasaj, Tharangrut Hanprasertpong, Savitree Pranpanus

**Affiliations:** 1 Department of Obstetrics and Gynecology, Faculty of Medicine, Prince of Songkla University, Hat Yai, Songkhla 90110, THAILAND

**Keywords:** Ultrasound, Fetal nasal bone, First trimester, Reliability, Reproducibility

## Abstract

**Background:**

Nasal bone assessment has been incorporated into Down syndrome screening in first trimester. Several studies have established the normal reference values for fetal nasal bone length in the first trimester, which were found to be varied by population. However, the study on reliability of nasal bone length measurement was limited with contradictory results. This study aimed to investigate the reliability of fetal nasal bone length measurement at 11–14 weeks of gestation in the Thai population.

**Methods:**

A total of 111 pregnant women at 11–14 weeks of gestation attending for the routine first-trimester ultrasound examination were recruited. Each case was measured separately by two examiners. Examiner 1 performed the first measurement in all cases; any of the other 5 examiners consecutively performed the second measurement. Three independent measurements were performed by each examiner and they were blinded to the results of the others. Intraobserver and interobserver variabilities were evaluated with the intraclass correlation coefficient (ICC).

**Results:**

Nasal bone measurement was successfully performed in 106/111 cases (95.5%) by at least one examiner; 89 cases were performed by two examiners. The intraobserver variability was excellent for all examiners (ICC, 0.840-0.939). The interobserver variability between different pairs of examiners varied from moderate to excellent (ICC, 0.467-0.962). The interobserver variability between examiner 1 and any other examiner was good (ICC, 0.749). The Bland-Altman plot of the interobserver differences of nasal bone length measurements between examiner 1 and any other examiner showed good agreement.

**Conclusions:**

The reliability of the fetal nasal bone length measurement at 11–14 weeks of gestation was good. The nasal bone length measurement was reproducible. Ethnicity has an effect on fetal nasal bone length, but reliability of nasal bone length measurement is critical to accuracy of screening and should be audited on an ongoing basis.

## Background

The first-trimester screening for Down syndrome is currently based on the combination of maternal age, nuchal translucency (NT) measurement, and maternal serum biochemical screening with a detection rate of 85%-90% for a 5% false positive rate [1-3]. As one of the most common characteristics of Down syndrome is a flat facial profile with a small nose, as described by Langdon Down in 1866 [4], the evaluation of the fetal nasal bone has been proposed to be incorporated into the Down syndrome screening.

According to the original study by Cicero et al. [5] evaluating the use of fetal nasal bone in screening for trisomy 21, an absent fetal nasal bone at the 11-14-week scan was found in 73% of trisomy 21 fetuses and in only 0.5% of chromosomally normal fetuses, which generated a strongly positive likelihood ratio of 146 (95% confidence interval (CI), 50–434) for absent nasal bone and 0.27 (95% CI, 0.18-0.40) for present nasal bone. They concluded that the incorporation of fetal nasal bone assessment into the first-trimester screening could result in a major reduction in the need for invasive testing and a substantial increase in sensitivity. It was estimated that screening for Down syndrome by combining maternal age, NT, maternal serum biochemical screening, and examination of the nasal bone could increase the detection rate to 97% with a false positive rate of 5% [6].

The effect of ethnicity on nasal bone length has been reported [7-10]. For example, a higher incidence of nasal bone hypoplasia was reported in the women of African-Caribbean origin than the Caucasian women [7]. Though the nasal bone length measurement in the first trimester for Down syndrome screening has been of limited use due to insignificant differences between affected and normal fetuses according to the study of Cicero et al. [11], several studies have concerned about racial differences and established a normogram of nasal bone length in their population for future studies. Reference values of nasal bone length in normal fetuses varied by population. We were also interested in the study on the reference values of nasal bone length in our population. Since nasal bone is a small bifid structure and is quite difficult to identify by ultrasonography, the reproducibility of fetal nasal bone measurement might be problematic. Therefore, before conducting a study on the reference values of nasal bone length, it is essential to conduct the study on the reliability of fetal nasal bone length measurement. Few studies have dealt with this scope with contradictory results. Some studies have shown only fairly reproducible results [12,13], while others have shown good intraobserver and interobserver reproducibility [7,14]. The aim of the study was to assess the reliability of fetal nasal bone length measurement at 11–14 weeks of gestation in the Thai population.

## Methods

The study was conducted after approval by the Ethics Committee of the Faculty of Medicine, Prince of Songkla University, at the Maternal Fetal Medicine Unit, Songklanagarind Hospital, a tertiary care center in Southern Thailand, during a period of three months. Women with a singleton pregnancy, who requested for routine first-trimester ultrasound examination at 11–14 weeks of gestation, were asked to participate in this study by the nurses at the antenatal clinic who were not involved in the research study. A total of 112 patients gave their written informed consent and were subsequently enrolled.

There were six examiners in this study. All of them had at least a 2-year experience in first-trimester screening. Before starting the project, all examiners were standardized and underwent a one-month intensive training period for nasal bone length measurement with audit and feedback. The fetal crown-lump length (CRL), nuchal translucency (NT), and nasal bone length were measured. The NT measurement was performed according to the method described by Nicolaides et al. [15].

All examiners were standardized for fetal nasal bone length measurement by the following protocol based on the study of Cicero et al. [16],

1. The image should be magnified so that each increment in the distance between the calipers was only 0.1 mm.

2. A mid-sagittal view of the fetal profile in the supine position should be obtained.

3. The face of transducer should be roughly parallel to the skin over the nasal bridge. In this position, the angle between the ultrasound transducer and an imaginary line passing through the fetal profile should be about 45 degrees.

**Figure 1:**
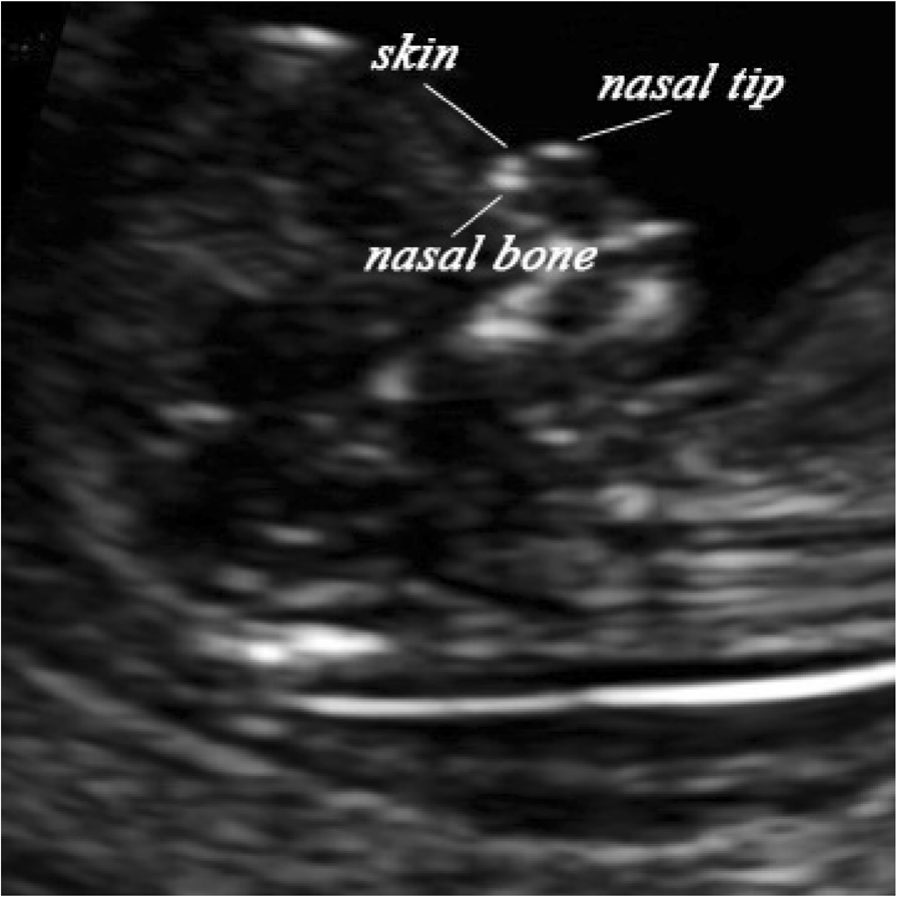
Ultrasound image in the midsagittal view showing nasal bone, nasal skin and tip of nose.

When all these criteria were met, the probe was moved from side to side to ensure that the nasal bone could be seen separately from the nasal skin. The skin over the nasal bridge, the nasal bone, and the cartilaginous tip of the nose were separately identified before measuring the nasal bone, which appeared as three distinct lines. Two of them, proximal to the forehead, were parallel to each other, resembling an “equal sign”. The upper one represented the skin and the bottom one which was more echogenic and thicker represented the nasal bone. The third one, at a higher level of the skin, represented the tip of nose (Figure 1). After the nasal bone was clearly apparent, the bony part of the nasal bridge was measured at the level of synostosis by placing the calipers in the out-to-out position. An absent nasal bone was diagnosed when the nasal cartilage line appeared as a thin and less echogenic line than the overlying skin. Due to the constraints of our clinic, the assessment of fetal nasal bone length was declared a failure if the total duration of scan took more than 30 minutes.

The ultrasonography was performed transabdominally in all cases by the Voluson 730 Expert (GE Healthcare, Kretztechnik, Zipf, Austria). The fetal images were stored on the hard drive of an ultrasound machine for subsequent audit. Each case was scanned separately by two examiners. Examiner 1 determined the presence of fetal nasal bone and performed the first measurement of the nasal bone length in all cases; any of the other five examiners consecutively performed the second measurement. Each examiner was blinded to the results of the others. Three independent measurements were performed by each examiner and the values were used for the calculation of intraobserver variability. Their average values were used for the analysis of interobserver variability.

### Statistical analysis

The statistical analysis was performed using the SPSS version 15.0 software (SPSS Inc., Chicago, IL, USA). The intraclass correlation coefficient (ICC) was used to calculate the intraobserver and interobserver variabilities. Bland-Altman plots were generated to examine the mean differences between examiners (difference between two paired measurements plotted against the average between measurements) using the MedCalc v. 12.1.4 software (MedCalc Software, Mariakerke, Belgium).

## Results

During the study period, a total of 112 cases were enrolled in this study. One case was excluded due to absent nasal bone. The fetal nasal bone length measurement was successfully performed by at least one examiner in 106/111 of cases (95.5%). Measurement failure was due to unfavorable fetal position (n = 4) and maternal obesity (n = 1). Eighty-nine cases (80.2%) were successfully performed by two examiners. Those cases with failure of measurement by one examiner (19.8%) were due to fetal position.

The median nasal bone lengths were 1.4 mm (range, 1.1-1.9), 1.7 mm (range, 1.1-2.5), and 2.1 mm (range, 1.5-2.6) at gestational age of 11, 12, and 13 weeks respectively. The median crown- rump length was 57.1 mm (range, 40.7- 75.9), and the median nuchal translucency thickness was 1.2 mm (range, 0.7-3.0). All cases had normal karyotype by prenatal diagnosis or normal phenotype at birth.

**Figure 2:**
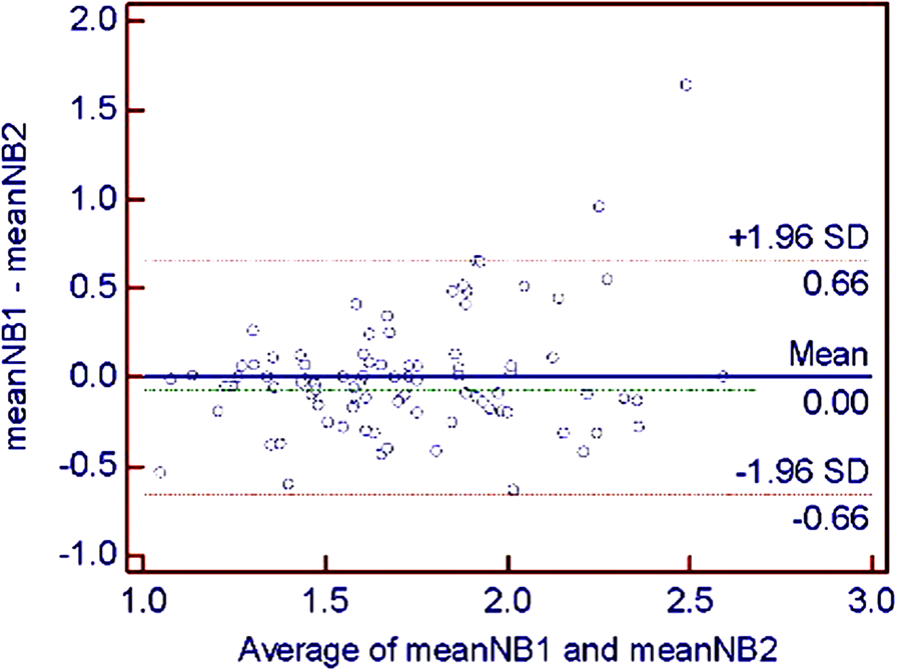
Bland-Altman plot of interobserver differences of nasal bone length measurement between examiner 1 and any other examiner.

Intraclass correlation coefficients for intraobserver variation were considered excellent for all 6 examiners as shown in Table 1. The interobserver variability varied from moderate to excellent between the different pairs of examiners as shown in Table 2, but the overall interobserver variability between examiner 1 and the other examiners was good. The Bland-Altman plot of the interobserver differences of nasal bone length measurement between examiner 1 and any other examiner shows a small mean difference with good agreement (Figure 2). Two cases of disagreement were related to maternal obesity.

**Table 1:** Intraclass correlation coefficients for each observer

**Examiners**	**Intraclass correlation coefficients**	**95% Confidence interval**
1	0.840	0.787-0.884
2	0.939	0.894-0.968
3	0.929	0.819-0.979
4	0.884	0.745-0.958
5	0.882	0.724-0.960
6	0.937	0.875-0.972

**Table 2:** Intraclass correlation coefficients between observers

**Examiners**	**Intraclass correlation coefficients**	**95% Confidence interval**
1 versus 2	0.920	0.835-0.961
1 versus 3	0.467	−0.981-0.857
1 versus 4	0.573	−0.399-0.870
1 versus 5	0.962	0.857-0.990
1 versus 6	0.931	0.830-0.972
1 versus any	0.749	0.618-0.836

## Discussion

Fetal nasal bone examination has recently been used as an additional screening tool in first-trimester Down syndrome screening. This study showed that intraobserver variability of fetal nasal bone length measurement at 11–14 weeks of gestation was excellent for all examiners, but interobserver variability varied among pairs of examiners, ranging from moderate to excellent. However, the overall interobserver variability between examiner 1 and any other examiner was good with an ICC of 0.749 and only a small mean difference and good agreement.

With regard to intraobserver variability, this study’s finding was consistent with those of previous reports [7,14]. Conflicting results mainly concerned interobserver variability, leading to an uncertainty of reproducibility of the fetal nasal bone measurement. For example, the study conducted by Senat et al. [12] reported only fair reproducibility of nasal bone identification, categorizing the results as present/uncertain/absent, by retrospective assessment of video-loops of a 30-second duration among three experienced operators with good agreement of only 80.7%, and the best Kappa value of only about 0.4. Bekker et al. [13] evaluated 90 cases between 11–14 weeks of pregnancy, divided into two equal sub-studies. Both were separated by an intensive 3-month training program for the operators. They found that the interobserver correlation in the first study was poor (inter-CC 0.32). Although, a slight improvement was noted (inter-CC 0.64), interobserver correlation was still moderate after the intensive training program. They concluded that the reproducibility of the fetal nasal bone length measurement in the first trimester was inadequate. Conflicting results were reported in the studies by Kanellopoulos et al. and Cossi et al., which demonstrated good intraobserver and interobserver variabilities [7,14].

Concerning interobserver variability in this study, five pairs of examiners with an experience of at least 2 years in first-trimester screening were evaluated. Though overall interobserver variability was good, each pair of examiners had different results varying from moderate to excellent depending on examiner. Moderate reproducibility was found in two pairs of examiners (1 versus 3 and 1 versus 4) and an excellent result in three pairs of examiners. Furthermore, standardization with audit and feedback was performed before conducting the study. We postulated that the differences were not solely related to interobserver variation. Technical factors and biological variation have to be considered in fetal nasal bone length measurement, as well as variation and interaction within examiners, subjects (pregnant women) and ultrasound image [8,9].

Regarding subject variation, maternal obesity, for example, has an effect on the quality of an ultrasound image, resulting in a higher chance of inaccuracy of nasal bone assessment, which leads to significant differences in measurement between examiners. Using high resolution ultrasound equipment is necessary to achieve good ultrasound images.

Proper training and standardization of the measurement technique with strict adherence to criteria are of importance in avoiding false results. The study of Cicero et al. [16] concerning the learning curve for sonographic examination of the fetal nasal bone at 11–14 weeks showed that the minimum number of scans required for an experienced sonographer to become competent in examining the fetal nasal bone was on average 80, with a range of 40–120. Since nasal bone is a small, bifid structure, obtaining an appropriate image is somewhat problematic. It can be easily missed if the image is not exactly in the midsagittal view, or the nasal bone is parallel to the ultrasound beam. Variance in nasal bone echogenicity and discrimination between echoes of nasal skin and bone lead to difficulties in nasal bone measurement even by an experienced sonographer.

Identification of fetal nasal bone is subjective. Technical and methodological errors may occur; for example, including or excluding the relatively hypoechogenic ends of nasal bone [17], measuring segments of the zygomatic bone instead of the nasal bone or including the tip of the nose in the nasal bone length measurement [10]. Methodological difficulties in nasal bone examination in the first trimester have been reported in the FASTER trial by Malone et al., in which well-trained sonographers who had little previous experience in evaluation of the nasal bone were able to depict the nasal bone in only 75.9% of 6316 fetuses [18].

In our study, satisfactory images from five cases were not obtained due to fetal position and maternal obesity. Difficulties in nasal bone examination experienced by our examiners were similar to those in nuchal translucency measurement, but nuchal translucency could be measured in either the prone or supine position of the fetus. Therefore, a higher failure rate is expected in nasal bone measurement unless more time is spent on waiting for the fetus to change the position. Transvaginal ultrasonography may be an option in cases of measurement failure. However, due to Thai culture characteristics, most women denied its use unless strongly indicated.

In order to improve the accuracy of nasal bone assessment, regular audit and feedback is mandatory. An abnormally large measurement should prompt a review of sonographic technique [17]; it is likely to be due to an incorrect technique of measurement rather than the fetus truly having a long nasal bone.

## Conclusions

This study showed excellent intraobserver variability and good interobsever variability with good agreement of fetal nasal bone measurement at 11–14 weeks of gestation among six examiners. Fetal nasal bone length measurement is reproducible.

## Abbreviations

NT: Nuchal translucency; CI: Confidence interval; CRL: Crown-lump length; ICC: Intraclass correlation coefficient.

## Competing interests

The authors declare that they have no competing interests.

## Authors’ contributions

CS carried out the research proposal development, nasal bone measurement, data analysis and drafted the manuscript. NP participated in the research proposal development, data collection, nasal bone measurement and edited the manuscript. OK, TS, TH and SP contributed in research conception, carried out the nasal bone measurement and edited the manuscript. All authors read and approved the final manuscript.

## Authors’ information

CS (MD, M.Med.Sci (Obstetric Ultrasound)) is an associate professor working as a specialist in Maternal Fetal Medicine. NP (MD) is a specialist in Maternal Fetal Medicine. OK(MD) is a professor and specialist in Maternal Fetal Medicine. TS (MD) is an associate professor and specialist in Maternal Fetal Medicine. TH (MD) is an assistant professor and specialist in Maternal Fetal Medicine. SP(MD) is a specialist in Maternal Fetal Medicine.

## Pre-publication history

The pre-publication history for this paper can be accessed here:

http://www.biomedcentral.com/1471-2393/13/7/prepub
